# Environmental Influences of Extreme Heat on the Health of Older Adults: A Retrospective Study

**DOI:** 10.1093/geroni/igaa057.897

**Published:** 2020-12-16

**Authors:** Mei Liu, Carol Buller, Barbara Polivka, Terri Woodburn, Mark Jakubauskas, Benjamin Wolfe

**Affiliations:** 1 University of Kansas, Kansas City, Kansas, United States; 2 University of Kansas Edwards Campus, Overland Park, Kansas, United States; 3 University of Kansas, Overland Park, Kansas, United States

## Abstract

Studies have suggested that extreme weather events have differential effects by age. By leveraging electronic medical records, we aim to analyze the environmental influence of extreme heat on the health of older adults. From our healthcare system’s de-identified data warehouse, we extracted a retrospective cohort of 108,192 patients who were ≥65 years of age as of 1/1/2018 with pre-existing chronic conditions including diabetes, COPD, cardiovascular disease, or kidney disease. Extreme heat event period was defined as 5/1/2018 to 9/1/2018 (79 days with temperature ≥90o; 15 days of moderately poor/poor air quality index (AQI) [≥75] values) and the comparison period was defined as 5/1/2019 to 9/1/2019 (51 days with temperature ≥90o; 0 days with moderately poor/poor AQI values) in the Kansas City area. We randomly partitioned the study cohort into two sets and demonstrated the two patient sets were statistically similar (p>0.05) with respect to their demographic and underlying health conditions. Finally, we compared the respiratory, cardiovascular, and renal health outcomes between the 2018 and the 2019 cohorts. Most patients were Caucasians, female and had comorbid conditions. Results showed significantly higher number of all-cause emergency department visits (p=0.04) and outpatient visits (p=<.001) during the extreme heat event period in 2018. Analyses also showed significantly higher number of outpatient visits due to upper respiratory diseases (p=0.008) and acute renal failure (p=0.01) in 2018. In conclusion, extreme heat increased use of healthcare services in older adults with chronic conditions.

